# aMMP-8 Oral Fluid PoC Test in Relation to Oral and Systemic Diseases

**DOI:** 10.3389/froh.2022.897115

**Published:** 2022-06-10

**Authors:** Timo Sorsa, Solomon Olusegun Nwhator, Dimitra Sakellari, Andreas Grigoriadis, Kehinde Adesola Umeizudike, Ella Brandt, Mutlu Keskin, Taina Tervahartiala, Pirjo Pärnänen, Shipra Gupta, Ritin Mohindra, Nagihan Bostanci, Nurcan Buduneli, Ismo Tapani Räisänen

**Affiliations:** ^1^Department of Oral and Maxillofacial Diseases, Head and Neck Center, University of Helsinki and Helsinki University Hospital, Helsinki, Finland; ^2^Section of Oral Health and Periodontology, Division of Oral Diseases, Department of Dental Medicine, Karolinska Institutet, Solna, Sweden; ^3^Department of Preventive and Community Dentistry, Obafemi Awolowo University, Ile-Ife, Nigeria; ^4^Department of Preventive Dentistry, Periodontology and Implant Biology, Dental School, Aristotle University of Thessaloniki, Thessaloniki, Greece; ^5^424 General Army Hospital, Thessaloniki, Greece; ^6^Department of Preventive Dentistry, Faculty of Dental Sciences, College of Medicine, University of Lagos, Lagos, Nigeria; ^7^Oral and Dental Health Department, Altinbaş University, Istanbul, Turkey; ^8^Oral Health Sciences Centre, Post Graduate Institute of Medical Education and Research, Chandigarh, India; ^9^Department of Internal Medicine, Post Graduate Institute of Medical Education and Research, Chandigarh, India; ^10^Department of Periodontology, Faculty of Dentistry, Ege University, Izmir, Turkey

**Keywords:** aMMP-8, point-of-care (PoC), biomarker, periodontitis, peri-implantitis, systemic diseases

## Abstract

The manuscript uses the previously published literature and highlights the benefits of active-matrix metalloproteinase (aMMP)-8 chairside/point-of-care (PoC) diagnostic tools as adjunctive measures in oral and systemic diseases. Previous studies suggest that as a biomarker, aMMP-8 is more precise than total MMP-8, MMP-9, MMP-2, MMP-3, MMP-13, MMP-7, MMP-1, calprotectin, myeloperoxidase (MPO), human neutrophil elastase (HNE), tissue inhibitor of matrix metalloproteinase (TIMP)-1, and bleeding of probing (BOP). Therefore, aMMP-8 could be implemented as the needed key biomarker for the new disease classification for both periodontitis and peri-implantitis. With a sensitivity to the tune of 75–85% and specificity in the range of 80–90%, lateral flow aMMP-8 PoC testing is comparable to catalytic protease activity assays for aMMP-8. The test can be further applied to estimate the glycemic status of an individual, to ascertain whether a person is at risk for COVID-19, in managing the oral side effects of radiotherapy carried in head and neck cancers, and in selected cases pertaining to reproductive health. In the future, aMMP-8 could find application as a potential systemic biomarker in diseases affecting the cardiovascular system, cancers, bacteremia, sepsis, diabetes, obesity, meningitis, as well as pancreatitis. The aMMP-8 PoCT is the first practical test in the emerging new dental clinical field, that is, oral clinical chemistry representing oral medicine, clinical chemistry, peri-implantology, and periodontology.

## Introduction

Matrix metalloproteinase-8 (MMP-8), collagenase-2, or neutrophil collagenase, identified in 1968 in human neutrophils and gingiva, were cloned from neutrophils from a granulocytic leukemia patient [1]. Its degranulation and expression can be induced by various microbial virulence factors, proinflammatory cytokines, such as interleukins and tumor necrosis factor-α, and CD40-ligand [2]. Glycosylation increases the size of mature MMP-8 from 60–70 kDa to 75 kDa. Autoproteolytic degradation and fragmentation related to zymogen activation yielding 50 kDa active form or species and 20–40 kDa fragments have been described [1, 2]. Latent proMMP-8 can be activated by MMP-3, MMP-10, MMP-7, trypsins, serine proteases, and microbial proteases as well as by oxygen-derived free radicals [3–9]. MMP-8 is expressed at the myelocyte phase during neutrophil development in the bone marrow, and stored and prepacted as latent MMP-8 in subcellular neutrophil-specific granules to be the degranulated and released extracellularly at the sites of inflammation in gingiva during periodontitis and peri-implantitis [10, 11].

## aMMP-8 Oral Fluid PoC Test in Relation to Oral Diseases

In periodontitis and peri-implantitis, MMP-8 is regulated as inductive neutrophil degranulating stimuli and zymogen activation and subsequently inhibited rather than translationally by the *de-novo* expression [10, 11]. It is inhibited endogenously by tissue inhibitors of matrix metalloproteinases (TIMPs) or pharmacologically by doxycycline and chlorhexidine [10–16]. MMP-8 cleaves not only type I–III collagens and various extracellular matrix (ECM) proteins but also non-matrix bioactive molecules such as serpins, bradykinin, angiotensin I, fibrinogen, insulin-receptor, and pro- and anti-inflammatory cyto- and chemokines involved to regulate immune and endocrinological processes [2, 10, 17]. MMP-8 is the most prevalent collagenolytic protease in the diseased periodontium and peri-implantium [2, 18–20]. Active MMP-8 (aMMP-8) is the major mediator of periodontitis and peri-implantitis tissue destruction [2, 6, 8, 10, 18]. The reduction of MMP-8 was shown to reduce periodontitis and peri-implantitis [2, 6, 8, 10, 18, 21].

To manage the periodontal and peri-implantological diagnostic challenges, dentists and periodontists are recommended to acquire the shift from the classical clinical, radiographic, and timely microbiological measurements to periodontal, peri-implant, tissue destruction proinflammatory biomarkers, and oral fluid metabolomics, as part of a new clinical field in oral medicine, that is, oral clinical chemistry [22]. The recent innovations of oral fluid [gingival crevicular fluid (GCF), peri-implant sulcular fluid (PISF), saliva, and mouthrinse], chairside, bedside, and point-of-care diagnostic (PoCT) technologies and kits enable this to be more versatile and practical in everyday work on the tissue destructive periodontal and peri-implant diseases [19, 23]. In this regard, the neutrophil collagenase, MMP-8, or collagenase-2, and especially active/activated and fragmented forms or species (aMMP-8 species) [2, 10, 24] are promising point-of-care test biomarker candidates or technologies [25].

### The Sensitivity and Specificity of aMMP-8 (Neutrophil Collagenase-2) Point-of-Care Oral Fluid Tests (PoCT)

**Figure 1:**
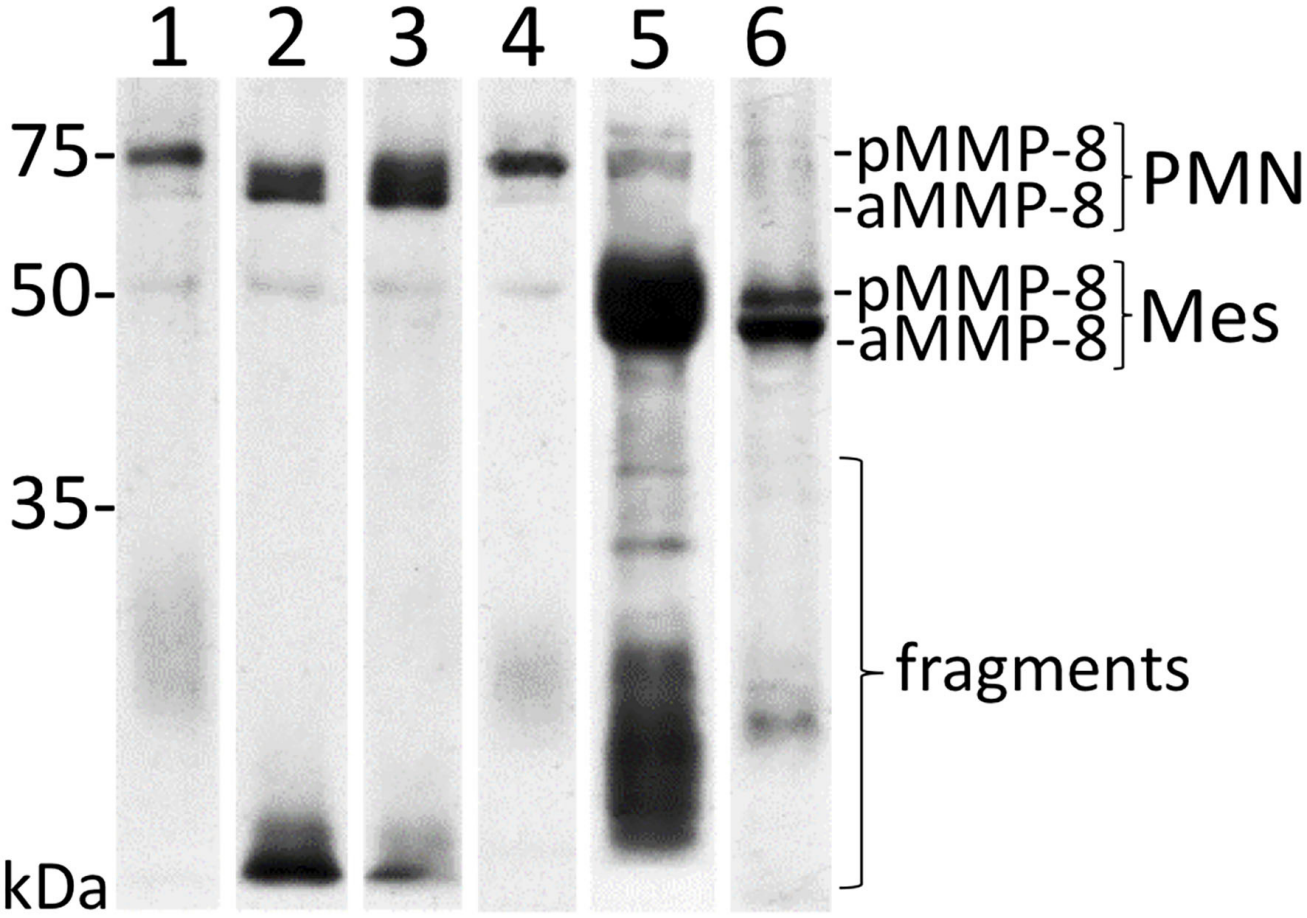
Representative Western immunoblot for molecular forms and species of MMP-8/collagenase-2 in human mouthrinse samples. Lane 1: recombinant human MMP-8, polyclonal antibody; lane 2: recombinant human MMP-8 activated by 200 μM NaOCl, polyclonal antibody; lane 3: periodontitis mouthrinse before treatment with polyclonal anti-MMP-8; lane 4: periodontitis mouthrinse after treatment with polyclonal anti-MMP-8; lane 5: periodontitis mouthrinse before treatment with monoclonal anti-MMP-8; lane 6: periodontitis mouthrinse after treatment with monoclonal anti-MMP-8. The p- and aMMP-8 (PMN and Mes) indicate neutrophil pro (p) and active (a) (PMN)- and fibroblast-type (Mes), MMP-8, respectively, and fragments indicate lower (<50 kDa) size MMP-8 species. Molecular weight markers are indicated on the left side.

The recently published studies have well-documented novel aMMP-8 chairside tests to be valid, reliable, reproducible, sensitive, and specific [2, 15, 20, 21, 26–29]. The results of these studies have established oral fluid aMMP-8 PoCT-test technology as a valid tool for addressing periodontal health and disease in both Caucasian adolescents and adults of Nigerian, Turkish, Indian, Chinese, Chilean, American, Greek, Malian, Italian, Finnish, and Swedish origin [15, 19, 21, 22, 25, 27–36]. The overall sensitivity and specificity of oral fluid aMMP-8 PoCT with a cut-off of 20 ng/mL for periodontitis and peri-implantitis in these studies have been found to be 76–90% and 85–96% depending on the definition of periodontal/peri-implant disease and health [19, 21, 33]. The technology was found to be highly valid when using mouthrinse, with the ability to detect poor oral hygiene with a sensitivity of 96%, bleeding on probing (BOP) from two sites with a sensitivity of 82.6%, and deep periodontal pockets in two or more areas of chronic periodontitis with a sensitivity of 95% [30]. Among adolescents, the aMMP-8 mouthrinse PoC test detected deep periodontal pockets (three sites or more) with a sensitivity of 76.5% and from the healthy controls (no deep periodontal pockets) with a specificity of 100% [33]. Using GCF, the aMMP-8 PoC test had a sensitivity of 83.9 % (Stage III and IV periodontitis and gingivitis) and specificity of 79.2 % (healthy controls) [23]. Furthermore, the aMMP-8 mouthrinse PoC test correlated well with patients' treatment needs (community periodontal index of treatment needs, CPITN) among both adults and adolescents [37]. One study used a cut-off of 10 ng/mL and presented a sensitivity of 33.2% and specificity of 93% for periodontitis; however, the same study also observed 89.7% sensitivity and 73.6% specificity for stage IV periodontitis when the aMMP-8 concentrations were adjusted by the number of teeth [36]. Thus, the aMMP-8 PoCT, but not total MMP-8 [24, 28, 32] analysis, is a convenient and practical adjunctive diagnostic tool to monitor the diagnosis treatment and medication as well as maintenance of both periodontitis and peri-implantitis [21, 25]. Periodontal and peri-implantitis diagnostic studies have consistently revealed an aMMP-8 analysis to be more exact than the estimation of total MMP-8 or other biomarkers [38]. Genetic polymorphisms of matrix metalloproteinase-3 (MMP-3) and vitamin D receptor (VDR) predispose an adolescent to periodontitis [12]. Heikkinen et al. [27] found the aMMP-8 chairside test to be beneficial in the identification of such individuals. After adjusting for smoking and visible plaque, radiographic findings characteristic of initial periodontitis were found to be positively correlated with TLR4 (rs498670) and TNFSF11 (rs2277438) [39]. Representative Western immunoblot shows the molecular forms and species MMP-8 detected in human oral fluid/mouthrinse samples by both polyclonal and monoclonal anti-MMP-8 antibodies (Figure 1); upon activation, latent proMMP-8 is converted to lower molecular size active forms/species and fragmented species that can be detected especially in the oral fluids/mouthrinse of the periodontitis and peri-implantitis patients [2, 6, 10, 13, 17, 19].

## aMMP-8 Oral Fluid PoC Test in Relation to Systemic Diseases

This novel aMMP-8 chairside test also finds applicability in establishing the relationships between oral, general, and systemic health parameters, and aMMP-8 oral fluid PoCT has also been investigated namely, reproductive health parameters [40–42], diabetes [43, 44], COVID-19-infection [45, 46], and head and neck cancers' radiotherapy and its oral destructive immunodystrophic side effects [47]. Umeizudike and associates recently highlighted the potential usefulness of the aMMP-8 biomarker PoCT as a bridge between oral/periodontal and systemic diseases [48].

### Periodontal, Reproductive, and Sexual Health and Concurrent aMMP-8 Levels

A potential link between periodontal health and systemic health including that of the reproductive system has existed for a long time. Although the Focal infection theory [49, 50], Focal allergy theory (Berger), and Focal toxicosis theory (Slauk) of the 1940's initially failed to gain acceptance [40], they were later conceptually reassessed by Linossier et al. [51]. The isolation of a sperm immobilizing factor from necrotic dental pulp *Escherichia coli* probably eventually sparked this renewed interest, but the investigators were neither dentists nor periodontologists, instead gynecologists [52]. Nevertheless, all this evidence remained uninvestigated for the next two decades [41].

In 1986, Bieniek and Riedel [52] addressed antibiotic-resistant bacteriospermia among patients with chronic periodontitis. No work was carried out on the relationship between fertility and oral health for over a decade till Offenbacher et al. [53] reported their findings on the association between chronic periodontitis and preterm birth. After this, a possible link between endometriosis and chronic periodontitis was reported by Kavoussi et al. [54]. Their work was followed up by studies suggesting a potential link between chronic periodontitis and sperm sub-motility [40, 41, 55]. In addition, Oguz et al. [56] and Eltas et al. [57] demonstrated an association between chronic periodontitis and erectile dysfunction as well as the beneficial effect of periodontal treatment. Furthermore, Hart reported a link between periodontitis and time to conception [58].

Although the exact relationship between periodontitis and reduced libido is still unclear, a plausible role of arginine has been reported, with this nitrous oxide precursor significantly influencing libido in both genders [59, 60]. Erectile dysfunction is characterized by the inability to attain or maintain a penile erection satisfactory for sexual intercourse. Arginine is essential for the achievement and maintenance of penile erection [61, 62]. Hence, any enhancement in the activity of the arginase enzyme could critically affect the male sexual arousal pathway [61–63]. *Porphyromonas gingivalis* induced enhanced arginase activity and hence could negatively affect the male libido [61, 62]. Higher levels of salivary arginase have been reported in chronic periodontitis patients, with a reduction in levels following periodontal therapy [64]. The arginine-*P. gingivalis* axis eventually can hence explain, at least partially, the link between chronic periodontitis and erectile dysfunction [65].

Endothelial dysfunction and injury caused by pro-inflammatory mediators of chronic periodontitis such as tumor necrosis factor-α (TNF-α), interleukin (IL)-1, and IL-6 could also affect the vasculogenic pathway resulting in erectile dysfunction [66–69]. These proposed mechanisms are still being investigated.

Lipid peroxidation with reduced total antioxidant capacity was found to be linked to low sperm count by Colagar and associates [70]. Camejo et al. reported raised IL-6 levels to be positively associated with lipid peroxidation, further supporting the possible chronic periodontitis-reduced sperm count link [71]. However, it is not clear whether elevated aMMP-8 levels in oral fluids could explain these problems. Nwhator et al. demonstrated reduced sperm counts to be significantly associated with the oral hygiene of an individual, though no such association was found between sperm count and chronic periodontitis [41]. However, it remains to be determined whether changes in male sexual potency are reflected in eventual changes in oral fluid aMMP-8 levels [72]. Further studies addressing oral fluid aMMP-8 point-of-care tests (PoCT) in relation to reproductive and sexual parameters are warranted.

### Periodontal Health, Conception, and Concurrent aMMP-8 Levels

Associations between endometriosis [54], pelvic inflammatory disease [73], and increased time to conception were first identified by Hart et al. [58] and later confirmed by Nwhator et al. [74]. Periodontitis has the potential to influence pregnancy outcomes in two ways: first, by releasing chronic pro-inflammatory mediators, or second by allowing periodontal pathogens direct access to the fetal circulation and amniotic fluid [75]. *Fusobacterium nucleatum* is a dysbiotic adhesive periodontopathogen that enhances the colonization of other dysbiotic periodontitis-associated bacteria [75, 76] and can transform into an “overt pathogen” by translocating to extraoral sites [77].

*F. nucleatum* was identified in the subgingival biofilm of a stillborn infant, whose mother had pregnancy-associated gingivitis [78]. This suggests evidence for the direct hematogenous dissemination of *F. nucleatum*. Experimental models have shown that *F. nucleatum* can traverse the endothelium and colonize the fetal-placental compartment via E-cadherin-binding FadA adhesin and TLR4-dependent necroinflammatory reactions [79].

In addition, *P. gingivalis*, the most important and key proteolytic dysbiotic periodontopathogen, can induce fetal loss through the production of cardiolipin-specific antibodies and the ability of gingipains to activate proMMP cascades including proMMP-8 and−9 [80, 81], whereas treatment with gingipain proteinase inhibitors prevented fetal death and preterm birth caused by *P. gingivalis* infection—eventually preventing at least partially proMMP [81].

The potential racial differences in the inflammatory cytokine levels and the prevalence of pre-term birth also attract attention. Anum et al. [82] suggested that proinflammatory cytokine genes and their receptors are associated with matrix metabolism, as the elevated cytokine levels increase the expression of the inductive and tissue destructive MMPs. Nonetheless, they were unable to determine the role of genetic variants in preterm birth between various populations [83].

The effects of cytokines, such as IL-6 and IL-10 in the pathogenesis of bone resorption in periodontitis, are influenced by their levels and interactions [84, 85]. Proinflammatory cytokines IL-1β, IL-6, and TNF-α are present in osteoclast precursor cells and mature osteoclasts, and it mediates bone resorption in periodontitis [85]. Without an imbalance of a relatively low level of the potent anti-inflammatory IL-10, elevated IL-6 levels alone may not account for the periodontal tissue damage [85]. Paalani et al. reported higher IL-6 levels among blacks than whites, but their study found no racial differences in TNF-, C-reactive protein, or IL-10 [84, 85]. Their findings can explain, at least in part, the observed elevated aMMP-8 levels in 84% of Nigerian black pregnant women assessed with a novel aMMP-8 PoCT/chairside qualitative test [42]. Nwhator et al. [42] pointed that the raised aMMP-8 levels may, at least in part, explain the differential preponderant preterm births in black women. This is supported by the reported effect of MMP-8 on the preterm premature rupture of membranes, intra-amniotic inflammation, and risk of an adverse pregnancy outcome [86]. Active MMP-8 in amniotic fluid is a powerful predictor of spontaneous preterm delivery [87], and an aMMP-8 assessment can have significant implications for preterm birth. The findings of Nwhator et al. [42] suggest that more research is needed to fully ascertain the potential racial disparities in preterm birth.

The underlying mechanisms between time to conception and chronic periodontitis have not been fully clarified [42, 58]. Although a direct relationship has been established between higher levels of tissue inhibitor of metalloproteinase (TIMP)-1 and successful fertilization-embryo transfer (IVF-ET) conception [83], further investigations are warranted to ascertain the implications of increased aMMP-8 levels.

To further investigate the link between reproductive health parameters and periodontitis, a novel aMMP-8 PoCT/chairside test was used to ascertain clinical conditions among men and women in Nigeria [30, 42]. Oral hygiene was shown to be significantly correlated with increased waiting time among non-pregnant black women attending Nigerian fertility clinics [42], and this merits further research.

### aMMP-8 Chairside PoCT Test in Respect to the Specific Periodontal Health Parameters in Pregnant Women

The widespread elevation of aMMP-8 in pregnant Nigerian black women, affecting over 90% of them, regardless of demographics, educational level, or trimester, was an unexpected finding [42]. Using a novel qualitative aMMP-8 PoCT chairside test, the researchers explored the probable link between chronic periodontitis and increased time to conception in 58 non-pregnant fertility clinic attendees trying for pregnancy and 70 pregnant controls. Periodontitis (i.e., visual aMMP-8 positivity > 20 ng/mL), as shown by the novel aMMP-8 chairside test, was positively linked with increased time to conception in their study [74].

The authors had earlier addressed the effects of chronic periodontitis on seminal fluid parameters using a novel aMMP-8 chairside test and reported a significant association between subnormal sperm count and poor oral hygiene across all age groups [41]. The sensitivity of the aMMP-8 test kit from the Nigerian study was 95% for periodontitis, 96% for poor oral hygiene, and 82.6% for bleeding on gentle probing [10]. All stated sensitivity values of the aMMP-8 test kit were for two sites with periodontal pockets or bleeding on gentle probing among adults corresponding well to previous studies [41, 55].

The authors earlier investigated the effects of chronic periodontitis on seminal fluid parameters using the new aMMP-8 chairside test and found a significant correlation between low sperm count and poor oral hygiene across all age groups [41]. According to the Nigerian study, the aMMP-8 test kit had a sensitivity of 95% for periodontitis, 96% for poor oral hygiene, and 82.6% for bleeding on gentle probing [10]. The aMMP-8 test kit's reported sensitivity values were for two sites with periodontal pockets or bleeding on gentle probing in adults, comparable to previous studies [41, 55]. As stated earlier, the use of the aMMP-8 PoCT/chairside test kit demonstrated increased aMMP-8 levels in 87% of pregnant Nigerian women [42]. This novel aMMP-8 chairside test kit also facilitated the detection of chronic periodontitis in association with a longer time to conception, and a link between low sperm count and poor dental hygiene [41]. Future research could be designed to expand on these findings.

### aMMP-8 as Part of a Screening Strategy for Prediabetes/Diabetes at the Dental Clinic

Type 2 diabetes mellitus (DMT2) has become a pandemic on a global scale, resulting in major morbidity, mortality, and financial implications for healthcare systems [88]. Yet, type 2 diabetes is often undiagnosed, since it is asymptomatic at the earliest stages of the disease. Prediabetes, defined as hyperglycemia, that is less than but close to the pathologic threshold [glycated hemoglobin A1c (HbA1c) 5.7–6.4%, and/or fasting plasma glucose (FPG) 100–125 mg/dL], almost always precedes type 2 diabetes but lifestyle interventions are effective in preventing its progression to diabetes [89, 90].

Given these facts, screening for these conditions, early detection, and intervention, particularly at the prediabetes stage, are critical for both patients and healthcare systems. Numerous studies have already explored the effectiveness of diabetes screening in dental clinics, applying various methods of patient selection and detection for hyperglycemia, and in this regard, a recent meta-analysis highlighted the need for additional research [91]. Furthermore, periodontitis is known to upregulate pro-inflammatory mediators such as tissue destructive matrix metalloproteinases (MMPs) in inflamed gingiva and oral fluids [92–94]. Among MMP members, MMP-8 and MMP-9 were found to be elevated in the blood of patients with type 2 diabetes and metabolic syndrome [95, 96], and higher concentrations of MMP-8 and−9 have been reported in the gingival tissues and oral fluids of diabetic patients with periodontitis [97]. Thus, MMP-8 could serve as a link between these two pathological conditions.

In a study in Greece, among 150 participants attending periodontal clinics, a chairside point-of-care (PoC) clinical strategy was applied to identify undiagnosed hyperglycemia [43, 97]. These patients were selected from a group of 731 participants who were at a high risk of developing diabetes mellitus (score > 9) according to the CDC-recommended self-assessed questionnaire (“Centres for Disease Control and Prevention, USA. Prediabetes Screening Test. National Diabetes Prevention Programme. Available at https://www.cdc.gov/diabetes/prevention/pdf/prediabetestest.pdf”).

Full-mouth clinical parameters of periodontal disease were recorded as part of a dataset including age, gender, smoking, education, and Body Mass Index (BMI). Chairside assessments included the determination of HbA1c levels with the Cobas^®^ b101 (Roche Diagnostics, Hoffmann La Roche, Mannheim, Germany) *in vitro* diagnostic test system and quantitation of aMMP-8 by the chairside/Point-of-Care (PoC) PerioSafe^®^ immunotest combined with the digital reader ORALyzer^®^ according to the manufacturer's instructions. Thirty-one patients out of the 150 tested were found to have unknown hyperglycemia (20.7%). No differences were observed in sex, education, a parent with diabetes, normal BMI, smoking, age ≥ 45 years, or prior diabetes testing between patients with HbA1c <5.7 and ≥ 5.7%, although subgroups differed in terms of BMI (kg/m^2^), tooth count, and percentages of 4 and 5 mm pockets. The diagnostic performance for HbA1c ≥ 5.7 was evaluated by receiver operator characteristic (ROC) curves and areas under the curve (AUC) for the following: age ≥ 45 years and BMI (AUC 0.651, *p* = 0.010); age ≥ 45years, BMI, and aMMP-8 (AUC 0.660, *p* = 0.006); age ≥ 45 years, BMI, and stage of periodontitis (AUC 0.711, *p* < 0.001); and age ≥ 45 years, BMI, aMMP-8, and stage of periodontitis (AUC 0.713, *p* < 0.001). The aMMP-8 biomarker was found to be the most optimal for chairside point-of-care online and real-time quantitative diagnostics for both diabetes and periodontitis at the dentist's office, outperforming other biomarkers such as total MMP-8, active and total MMP-9, TIMP-1, myeloperoxidase (MPO), human neutrophil elastase (HNE), and calprotectin [98].

According to the findings and consistent with the literature, participants with HbA1c > 5.7 exhibited statistically significant differences in terms of periodontal disease clinical parameters, which underscores the contribution of hyperglycemia to periodontal tissue inflammation. This fact was also demonstrated when the periodontitis stage according to the 2018 classification [99] was integrated into the Receiver Operator Curves for diagnosing hyperglycemia (HbA1c ≥ 5.7%) either in combination with age ≥ 45 years and BMI or in combination with the above and aMMP-8 values (ORALyzer^®^). Therefore, increasing stage and grade of periodontitis, increasing age, and splanchnic obesity, as well as elevated aMMP-8 levels in mouthrinse, appear to be important factors for a dentist to encourage their patients to screen for diabetes. In the case of the aMMP-8 PoCT test, its application in the dental office, especially for periodontitis patients, can strengthen a dentist's reason for a patient to be further evaluated by a physician and receive recommended instructions/treatment, while HbA1c assessment is not yet easily feasible in a dental practice.

Furthermore, the incorporation of validated biomarkers will improve the diagnostic accuracy and assessment of the stage and grade of the new periodontitis classification system by Tonetti et al. [100] and recent data indicate that the aMMP-8 mouthrinse test can provide this capability in periodontitis as well as peri-implantitis [21, 25, 28, 101, 102]. With this background, we now present a modified new classification table with aMMP-8 implemented as the needed biomarker for periodontitis and peri-implantitis (Table 1). This fact is supported by the present data, as the subgroup of participants with aMMP-8 levels above 20 ng/mL demonstrated statistically significant differences in clinical parameters of periodontal and peri-implant diseases when compared to subjects with PerioSafe^®^- and ImplantSafe^®^ ORALyzer^®^ aMMP-8 values below this threshold. The aMMP-8 PoCT-test values <20 ng/mL can be regarded as biomarkers of periodontal and peri-implant health [21, 25, 28, 101, 102].

**Table 1:** Periodontitisgrading classification with aMMP-8 implemented as the biomarker (Tonetti et al. [100] table modified by Sorsa et al. [25]^*^).

**Periodontitis grade**			**Grade A: Slow rate of progression**	**Grade B:Moderate rate of progression**	**Grade C: Rapid rate of progression**
**Primary criteria**	Direct evidence of progression	Longitudinal data (radiographic bone loss or CAL)	Evidence of no loss over 5 years	<2 mm over 5 years	≥2 mm over 5 year
	Indirect evidence of progression	% bone loss/age	<0.25	0.25 to 1.0	>1.0
		Case phenotype	Heavy biofilm deposits with low levels of destruction	Destruction commensurate with biofilm deposits	Destruction exceeds expectation given biofilm deposits; specific clinical patterns suggestive of periods of rapid progression and/or early onset disease (e.g., molar/incisor pattern; lack of expected response to standard bacterial control therapies)
**Grade modifiers**	Risk factors	Smoking	Non-smoker	Smoker <10 cigarettes/day	Smoker ≥10 cigarettes/day
		Diabetes	Normoglycemic /no diagnosis of diabetes	HbA1c <7.0% in patients with diabetes	HbA1c≥7.0% in patients with diabetes
**Risk of systemic impact of periodontitis**	Inflammatory burden	High sensitivity CRP (hsCRP)	<1 mg/L	1 to 3 mg/L	>3 mg/L
*Biomarkers	**Indicators of CAL/bone loss or collagen destruction**	**Mouthrinse, gingival crevicular fluid (GCF), peri-implant sulcular fluid (PISF)**	**No/Slow** **=** **aMMP-8 level** **<** **10 ng/mL (no) OR 10–19.9 ng/mL (slow)**	**Moderate** **=** **aMMP-8 level** **>** **20 ng/mL**	**Rapid** **=** **aMMP-8 level** **>** **30 ng/mL**

Taken collectively, these findings suggest that when chairside methods of HbA1c assessment are unavailable, the combination of periodontitis, increasing age, BMI, and aMMP-8 appears to be a viable screening strategy for correctly referring dental patients to their physicians for further prediabetes/diabetes testing.

### aMMP-8 as an Integral Part of Perio-COVID-19 Connection

As logic would dictate, the understanding of the COVID-19 pandemic has been based on a sound basis of already existing literature on viral infections and the immune response these entail to devise management strategies. Translating this into the perspective of periodontal disease, which happens to be researched and understood exhaustively both in terms of its pathophysiology and management, it became evident that several commonalities existed between the COVID-19 disease process and that of periodontal disease.

The most imperative and glaring of these commonalities seems to be that which relates to the cytokine elevation profile. As both these disease processes have as their underlying mechanism an exaggeration of the host immune response, it would seem appropriate to draw a connection between the elevated cytokine profiles, which both COVID-19 and periodontal disease would seem to exhibit. The cytokine profile demonstrated in COVID-19 has been touted to be responsible for its symptomatic presentation. Such a cytokine response, termed “cytokine storm,” presents as an elevation of IL-7, IL-1β, IL-17, IL-2, IL-9, IL-8, G-CSF, GM-CSF, TNF α, IFN-γ, MIP1B, MIP1A, IP10, and MCP1. Patients with a further exaggeration in host cytokine response have been observed to require ICU admission more often [103]. The Th17 variety of inflammatory responses as observed in cases of infections caused by SARS-CoV and MERS-CoV have been equated to those observed in COVID-19 patients with adverse presentations such as pulmonary tissue damage and lung edema. The inflammatory pathway of periodontal disease distinctly overlaps the one observed in COVID-19, which points toward a high degree of suspicion of these disease processes being closely related. This would not only imply the effect periodontal disease would have on the presentation of COVID-19 but would also aid to form a basis for emphasizing greater importance on the maintenance of oral hygiene as well.

Another pertinent pathophysiological mechanistic overlap between periodontal disease and COVID-19 relates to NETosis which involves the generation of decondensed chromatin in a web-like fashion which eventually primes neutrophils to eliminate these, leading to cell death [104]. This can cause damage by either acting directly or indirectly in the form of bystander damage, which is so often associated with periodontal disease as a means of causing tissue damage. Both COVID-19 and periodontal disease have been observed to demonstrate the impaired removal of these NETs which end up causing a constant stream of harm to the region in which they populate [104].

Establishing a strong basis in literature to make the case for the possibility of a relationship between periodontal disease and COVID-19, an investigation into the recovery of the SARS CoV-2 in the vicinity of the periodontal apparatus assumes significant value. The gingival crevicular fluid (GCF) has been repeatedly demonstrated to be representative of the serum status of patients in numerous studies pertaining to several pathophysiological processes. The GCF of patients suffering from SARS CoV-2 has been demonstrated to harbor the SARS CoV-2 RNA which incidentally forms the basis of nasopharyngeal swab sampling, which is widely regarded as the gold standard [105]. The sensitivity for GCF to demonstrate SARS CoV-2 RNA was observed to be 63.64% (CI = 45.1–79.60%) and was 64.52% for saliva (CI = 45.37–80.77%) [105]. This emphasizes a significant level of comparability between saliva and GCF in terms of both harboring the SARS CoV-2 RNA and consequentially, as a sampling methodology as well. Saliva samples inherently involve the presence of GCF, which begs attention to the degree to which periodontal disease may influence sampling results, and indeed the COVID-19 disease process as well [106].

GCF sampling and nasopharyngeal sampling tend to be tedious in certain cases while being altogether unamenable in patients with reduced or non-existent mouth opening such as those suffering from trismus due to a variety of causes, TMJ ankylosis, severely deviated nasal septums, exaggerated gag reflex, and anatomically exaggerated nasal spurs. To this end, buccal swabs have been espoused and demonstrated to be a viable option to the gold standard of nasopharyngeal swab sampling with the former demonstrating a sensitivity of 58.9% compared to 62.90% exhibited by saliva sampling [107].

The fact that the SARS CoV-2 has been isolated from every perceivable oral entity, such as saliva, GCF, plaque, and calculus, has aided in exploring alternative sampling methodologies. The toothbrush forms one such modality wherein SARS CoV-2 RNA was detected with a sensitivity of 60% which is comparable to the values reported for GCF (63.64%), saliva (64.52%), and buccal swabs (58.9%) [108]. Sampling by this means is economical, painless, and amenable to self-collection as well.

The seemingly inseparable relationship between the oral cavity and COVID-19 becomes further manifested in the light of the proven clinical association between the presence of periodontal disease and the occurrence of adverse COVID-19-related outcomes [109–112]. It has been observed that increased severity of periodontal disease led to a commensurate increase in the necessitation of ventilation (odds ratio 7.45), an odds ratio of 14.58 of succumbing to the disease, an odds ratio of 36.52 for admission to a hospital, and that of 4.42 of suffering from pneumonia-related to COVID-19 [109]. Correlating the cytokine profile of the two disease processes, one could argue that aMMP-8, an established biomarker for the detection of early stages of periodontal disease was also found to be raised in COVID-19 patients, may be utilized to detect at-risk patients [110]. The availability of chairside/bedside tests of both the mouthrinse and site-specific variant of the aMMP-8 kit make this an ideal diagnostic modality for its purpose. When applied to a patient cohort suffering from COVID-19 and related to the presence or absence of periodontal disease, the mouthrinse variant of the aMMP-8 kit was observed to demonstrate a slightly greater sensitivity but a lower specificity when compared to the site-specific version [109, 110]. The values rose further when adjusted for gender, age, and smoking status. This provides the basis for these kits being purposed as screening tools to detect the presence of periodontal disease in patients afflicted with COVID-19, the relevance of which has already been established with some credence.

With periodontal disease being increasingly recognized as an active player in the pathophysiology of several systemic diseases, there is sufficient evidence in the literature to implicate its role in the pathophysiology of COVID-19 as well. From data reported for periodontal disease and associated COVID-19-related adverse outcomes, it is evident that mortality in these patients was determined to a significant extent by the presence of periodontal disease [109, 110].

Not only this but patients suffering from periodontal disease have been observed to demonstrate longer periods of illness when afflicted by COVID-19 as was commensurate with the severity of the periodontal disease. Patients in a state of periodontal health demonstrated an average of 4.15 days of being affected by COVID-19, 5.76 days with gingivitis, and 7.37 days with periodontitis, [112]. This further emphasizes the importance of oral health in COVID-19-related outcomes as periodontal diseases seemingly affect every conceivable pathophysiological manifestation of COVID-19.

### Link Between aMMP-8 Levels and the Periodontal Tissue Destruction During Radiotherapy of Head and Neck Cancer Patients

Head and neck cancer (HNC) was globally the seventh most common cancer in 2018 with 890,000 new cases and nearly 450,000 deaths [113]. However, the survival among HNC patients has improved over the years thanks to the improved treatment modalities that are used for eliminating malignant tumor cells [114]. For most HNC patients, the standard treatment modality is radiotherapy either alone or together with other treatment options [114, 115]. Unfortunately, radiotherapy has several negative side effects, including those on the periodontium and the oral immune fitness, which increases the risk of progression of attachment loss and initiation and activation of periodontitis for these patients [47, 116–118]. Management of the oral health of HNC patients having radiotherapy treatment is therefore imperative. Particularly new diagnostic methods that are available for the early detection of tissue destruction and immune dysregulation of the periodontium during or induced by the radiotherapy may play a key role in the adjunctive screening of patients at risk.

**Figure 2:**
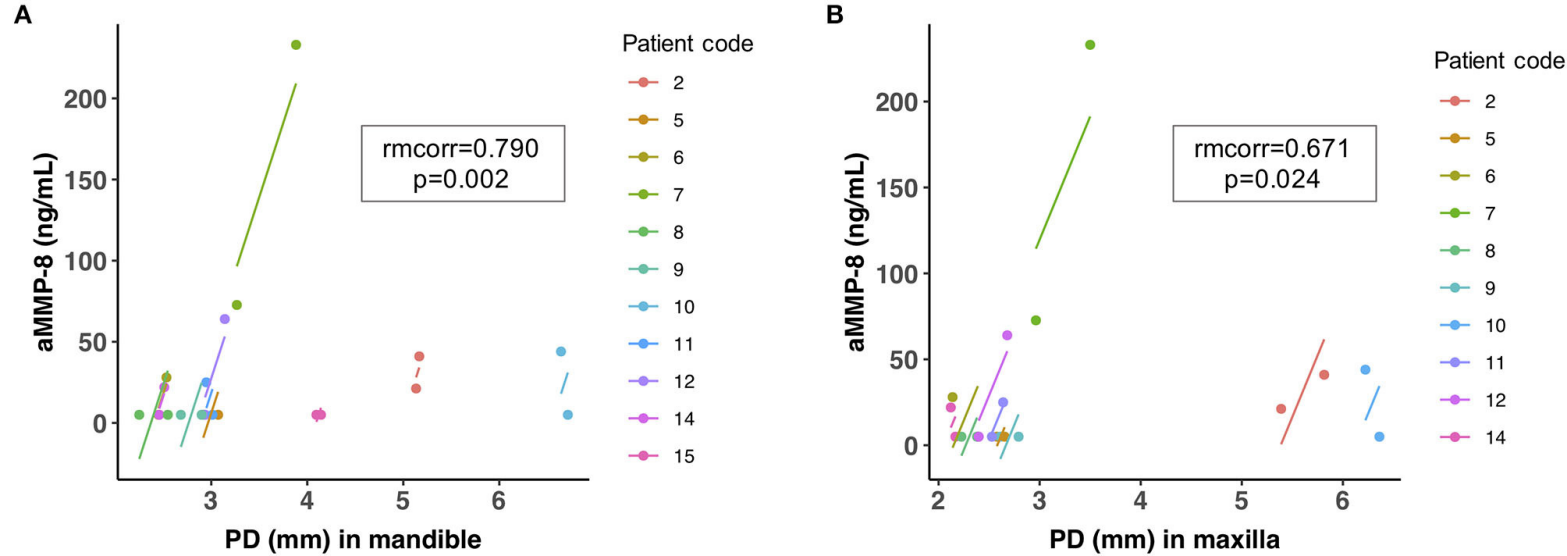
Repeated measures correlation (rmcorr) plot for significant positive correlation between aMMP-8 levels in mouthrinse and probing depth (PD, [mm]) in **(A)** mandible and **(B)** maxilla that were measured before radiotherapy and 1 month after the end of radiotherapy for 11 head and neck cancer patients as described in Keskin et al. [47].

A recent pilot study by our study group investigated two potential periodontal biomarkers, aMMP-8 and IL-6, in oral fluids (mouthrinse) of 11 HNC patients to identify and diagnose the negative side effects of radiotherapy on the periodontium [47]. We found that the radiotherapy treatment of patients was followed by a rapid progression of periodontitis measured by the clinical attachment loss (CAL), while the aMMP-8 levels measured by a point-of-care technology (PoCT) were significantly elevated after 6 weeks of radiotherapy and some HNC patients had elevated aMMP-8 levels even after 1 month at the end of the radiotherapy [47]. This suggests that aMMP-8 levels in mouthrinse can be a useful biomarker and chair/bedside test for quantitative online and real-time detection of the prolonged negative effect of HNC radiotherapy on the periodontium and the risk of further periodontal tissue destruction. Moreover, Figure 2 represents the repeated measures correlation between mouthrinse/oral fluid aMMP-8 levels and probing depth (PD) in both maxilla and mandible in the same Turkish cohort as in the study by Keskin et al. [47]. There was a significant positive repeated measures correlation in maxilla and mandible (rmcorr = 0.671, *p* = 0.024 and rmcorr = 0.790*, p* = 0.002, respectively) between aMMP-8 levels in mouthrinse and PD. In this regard, these results are in agreement with and further extend the prospective study by Lee et al. [119], which was one of the first to demonstrate a direct correlation between the progression of periodontal attachment loss (periodontal tissue destruction) and the elevation of aMMP-8 levels. Therefore, as Keskin et al. [47] stated, point-of-care/chairside oral fluid biomarker diagnostics, especially those based on aMMP-8, could benefit as an online and real-time preventive diagnostic tool for monitoring and quantitatively assessing the risk of active periodontal tissue destruction and attachment loss during HNC radiotherapy. Our findings also—for the first time—demonstrate that aMMP-8 PoCT is a quantitative online and real-time biomarker to be well-implemented in the new staging and grading classification of periodontitis in patients receiving radiotherapy for their head and neck cancer [47]. Noteworthy, aMMP-8 PoCT-technology can thus be well adapted to quantitatively real-time online and quantitatively chairside monitor and follow the outcomes of the emerging host modulating treatments [110, 120, 121]. Further studies are warranted in this area.

## Summary

Overall, aMMP-8 PoCT provides promising adjunctive quantitative diagnostic chairside/bedside technology and tool for oral as well as related systemic conditions. It can be adapted and implemented to monitor and follow the host modulation treatments and medications. Eventually, the test can be applied to the other medical fields including selected reproductive health parameters, cancers, cardiovascular diseases, diabetes, obesity, COVID-19/post-COVID-19, bacteremia, sepsis meningitis, and pancreatitis. The aMMP-8 PoCT is the first practical test in the emerging new dental clinical field, that is, oral clinical chemistry representing oral medicine, clinical chemistry, peri-implantology, periodontology, and medicine.

## Author Contributions

TS, NBo, IR, NBu, DS, SG, and PP contributed to the conception and design of the study. MK, IR, TT, EB, and TS contributed to data acquisition, analysis, data figures, and interpretation. TS, SN, DS, AG, KU, TT, SG, RM, and IR wrote the first draft of the manuscript. All authors contributed to manuscript revision, read and gave final approval, and agree both to be personally accountable for the authors' contributions and to ensure that questions related to the accuracy or integrity of any part of the work, even ones in which the authors were not personally involved are appropriately investigated, resolved, and the resolution documented in the literature.

## Funding

TS received financial support for this study from the Finnish Dental Society Apollonia, Finland; the Karolinska Institutet, Stockholm, Sweden; the Helsinki and Uusimaa Hospital District (HUS), Grant/Award Numbers: Y1014SULE1, Y1014SL018, Y1014SL017, TYH2019319, TYH2018229, TYH2017251, TYH2016251, and TYH2022225. The funders had no role in the design of the study; in the collection, analyses, or interpretation of data; in the writing of the manuscript, or in the decision to publish the results.

## Conflict of Interest

TS is the inventor of U.S. patents 1,274,416, 5,652,223, 5,736,341, 5,864,632, 6,143,476 and US 2017/0023571A1 (issued June 6, 2019), WO 2018/060553 A1 (issued May 31, 2018), 10,488,415 B2, and US 2017/0023671A1, Japanese Patent 2016-554676 and South Korean Patent No. 10-2016-7025378. The remaining authors declare that the research was conducted in the absence of any commercial or financial relationships that could be construed as a potential conflict of interest.

## Publisher's Note

All claims expressed in this article are solely those of the authors and do not necessarily represent those of their affiliated organizations, or those of the publisher, the editors and the reviewers. Any product that may be evaluated in this article, or claim that may be made by its manufacturer, is not guaranteed or endorsed by the publisher.
